# Diagnostic value and reliability of the present-on-admission indicator in different diagnosis groups: pilot study at a Swiss tertiary care center

**DOI:** 10.1186/s12913-018-3858-3

**Published:** 2019-01-09

**Authors:** Karen Triep, Thomas Beck, Jacques Donzé, Olga Endrich

**Affiliations:** 1 Medical Directorate, Inselspital, University Hospital of Bern, Bern, Switzerland; 2 Department of General Internal Medicine, University Hospital of Bern, Bern, Switzerland; 3 Direktion Medizin Insel Gruppe, Operatives Medizincontrolling Kodierung, University Hospital, Bern, CH-3010 Switzerland

**Keywords:** Present on admission, Routinely collected health data, Diagnostic value, Quality indicator, Complications and comorbidities, Quality of inpatient care

## Abstract

**Background:**

With few exceptions the International Statistical Classification of Diseases (ICD) codes for diagnoses and official coding guidelines do not distinguish pre-existing conditions from complications or comorbidities which occur during hospitalization. However, information on diagnosis timing is relevant with regard to the case’s severity, resource consumption and quality of care. In this study we analyzed the diagnostic value and reliability of the present-on-admission (POA) indicator using routinely collected health data.

**Methods:**

We included all inpatient cases of the department of medicine during 2016 with a diagnosis of deep vein thrombosis, decubitus ulcer or delirium. Swiss coding guidelines of 2016 and the definitions of the Swiss medical statistics of hospitals were analyzed to evaluate the potential to encode information on diagnosis timing. The diagnoses were revised by applying the information present-on-admission by a coding specialist and by a medical expert, serving as Gold Standard. The diagnostic value and reliability were evaluated.

**Results:**

The inter-rater reliability for POA of all diagnoses was 0.7133 (Cohen’s kappa), but differed between diagnosis groups (0.558–0.7164). The rate of POA positive of the total applied by the coding specialist versus the expert was similar, but differed between diagnoses. In group “thrombosis” SEN was 0.95, SPE 0.75, PPV 0.97 and NPV 0.60, in group “decubitus ulcer” SEN 0.89, SPE 0.82, PPV 0.89 and NPV 0.82, in group “delirium” SEN 0.91, SPE 0.65, PPV 0.71 and NPV 0.88 For all diagnoses SEN 0.92, SPE 0.73, PPV 0.87, NPV 0.82, summing up the cases of all diagnosis groups.

**Conclusions:**

Coding the POA indicator identified diagnoses which were pre-existent with insufficient reliability on individual patient’s level. The overall fair to sufficient diagnostic quality is appropriate for screening and benchmarking performance on population level. As the medical statistics of hospitals carries no variable on pre-existing conditions, the novel approach to apply the POA indicator to diagnoses gives more information on quality of hospital care and complexity of cases. By preparing documentation for POA reporting diagnostic quality must be increased before implementation for risk-assessment or reimbursement on the individual patient’s level.

**Electronic supplementary material:**

The online version of this article (10.1186/s12913-018-3858-3) contains supplementary material, which is available to authorized users.

## Background

The encoded data of inpatient stays in Switzerland are submitted to the Federal Office of Statistics (BFS) on an annual basis for publication of epidemiological and economic health care statistics. Furthermore, they allow the classification of cases for reimbursement of acute inpatient care into Swiss Diagnosis Related Groups (Swiss DRG). Although the Swiss medical statistics of hospitals contains ICD-10 German Modification 2014 (ICD-10 GM 2014) diagnoses including flags for laterality and secondary codes for causation it gives no information on diagnosis timing [1, 2]. Coding guidelines of 2016 in Switzerland include rules for encoding primary and secondary diagnoses as well as complications. Administrative data are increasingly used for disease surveillance, research, quality monitoring, case-mix costing (e.g. Swiss DRG), tracking healthcare performance and policy-making. Not only the publication of quality indicators by the Federal Office of Health (BAG) but also arising discussions on performance-based payment proposals show the need of a valid database [3–6]. A variable for diagnosis timing has been introduced as “diagnosis onset type” in Australia, “present-on-admission” in the United States of America (US), “diagnosis type” Canada and recently as “present-on-admission” in Austria [6, 7]. It is internationally recommended by the Word Health Organization (WHO) [3] and on national scale by Initiative Qualitätsmedizin (IQM) [8].

On behalf of the Centers for Medicare and Medicaid Services (CMS) in the US health care system the POA variable has to be reported since 2008. With effect on case-mix calculation it relates to hospital reimbursement claim [9]. The introduction in Switzerland would be consistent with aims in national health care policy concerning quality [10]. The experience gained elsewhere could serve the Swiss health care system to prepare the introduction of the variable POA into the Swiss medical statistics of hospitals. It is mandatory that quality measures accompany the introduction of SwissDRGs [11]. The variable seems suitable for population based models or risk adjusted algorithms of quality monitoring [12, 13]. In Europe some health systems enhance quality monitoring partially to support the introduction of reimbursement by DRGs in order to avoid adverse effects [5, 14, 15]. In Germany and Switzerland the Initiative Qualitätsmedizin e.V (IQM) supplies quality indicators derived from routinely collected health data and peer reviews are initiated selecting cases for review using ICD coding [16]. In Switzerland the Federal Office of Public Health publishes quality indicators for inpatient stays annually also derived from routinely collected data [17]. The POA indicator could be used to further develop the indicators in order to ensure quality of care. With public provision (e.g. Scandinavian countries, Spain) and central financing (e.g. Italy central financing but regional management) economical pressure is lower [15]. Nevertheless monitoring of health care indicators is implemented (e.g. Organisation for Economic Co-operation and Development OECD health care quality indicators including patient safety indicators) [18]. International comparisons should take into account that there are variations between member states with regard to data sources and calculating of indicators. Some OECD member states use register data, some population surveys. Thus, data are collected as samples or from certain regions only. The OECD guidelines define that the data for several indicators should be based on diagnosis on admission. In some European countries quality standards are part of negotiations between insurers and hospitals and part of global payment for all patients rather than for reimbursement on individual patients’ level (i.e. Germany, the Netherlands) [15, 19].

The resources of administrative data are limited. There are certain requirements to report on the complications of inpatient stays according to the Swiss coding guidelines, but both coded information on complications and other diagnoses do not sufficiently distinguish between being present at time of admission or not [20]. As there is increasing interest in using routinely collected health data, the at present limited information on the time of onset needs investigation [3, 5, 14, 21].

A survey conveyed by the Bern University of Applied Sciences, Bern, Switzerland, has demonstrated that different institutions and healthcare providers in Switzerland judge the current situation as insufficient. According to the results of the survey outcome analyses based on administrative data should be improved. Generally, valid information on the health status of a hospital’s patient population as well as on the hospital’s quality of care seems essential [22–24], (unpublished data, see Additional file 1). The survey showed a high interest in quality monitoring and benchmarking by further developed administrative datasets.

By implementing the indicator POA in different health care systems worldwide the utility of administrative data for risk-adjustment could be improved [5, 21, 25, 26], depending on the diagnostic quality and validity [24, 25, 27–31]. Nevertheless, during the years since introduction in the United States different studies and reports aiming on validity, reliability and diagnostic value have shown a diversity of results depending on the type of hospital, on the diagnosis itself and on measures of improving documentation and also revealed problems of over-reporting [23, 24]. Partially to avoid overreporting transparency has been introduced by publishing reports on case revisions [32] and quality indicators [16] in Germany and Switzerland. Automated screens for the plausibility of present-on-admission were developed to assess the accuracy of POA coding. The rate of secondary diagnoses, acute exacerbations, chronic diseases related to high-risk admissions, elective surgery or mortality derived from routinely collected data serve as a cost efficient method. Furthermore, with a set of statistical tests precision, bias, and validity can be assessed (e.g. variation, R-squared, correlation) [33, 34]. Still, the reliability concerning different diagnosis groups has not been tested so far, but is relevant in respect to measures implemented consecutively. The variable POA is dependent on the prevalence of the underlying conditions and diagnoses and the associated coding guidelines as it is always applied additionally to a certain ICD code. Prevalence can be estimated using ICD codes coded in a hospital’s or a national dataset. With different national coding guidelines the prevalence of diagnoses differ, and subsequently that of the POA indicator. By Swiss coding standard inpatient cases are coded after the patient’s discharge retrospectively. Only those diagnoses which could be confirmed and caused resource consumption during the inpatient stay are allowed to be coded [20].

Using administrative data based on ICD coding without knowledge of regulations might lead to misinterpretation as “In the absence of such a flag of diagnosis timing, researchers often assume that the main condition was present on admission, and then treat conditions coded in the secondary diagnosis fields as comorbidities for risk-adjustment.” [4, 6].

In order to introduce the variable POA to assess the quality of care and the complexity of admitted patients the diagnostic quality has to be evaluated. The study we performed aimed on analyzing retrospectively the diagnostic quality and reliability of the indicator present-on-admission of a limited selection of diagnoses encoded in cases of the Department of General Internal Medicine, University Hospital of Bern, Switzerland in 2016. The applied indicator POA was validated by medical expertise based on the complete medical record. The medical expert served as Gold Standard as within the Swiss coding regulations the medical judgement and documentation serves as the final verification. Coders themselves are not allowed to derive diagnoses from findings and when disparities occur the final authority will be the physician involved in the patient’s treatment. Although medical experts’ judgement on diagnoses and findings vary and the reliability can be questioned as well, within the Swiss coding system they serve as final instance [20].

The aim of the study was first to show that by attributing the indicator POA more information on the assessment of diagnosis timing can be obtained from administrative data compared to the limited requirements of the Swiss medical statistics of hospitals of 2016, second to analyze the diagnostic value and reliability of the indicator when encoded by coding specialists compared to the judgement of a medical expert and third to demonstrate the reliability and validity of POA when applied to different diagnosis groups.

An assessment at the Inselspital Bern with over 60.000 discharges annually showed, that for applying the POA indicator in addition to each coded ICD diagnosis (450.000 per year) would consume a budget of 1600 working hours. To reduce the load only specific diagnoses serving as indicators for quality could be chosen. The implementation of POA and using POA as a quality indicator or even as a factor in reimbursement (pay-for-performance) in different European countries needs a thorough discussion which should be based on studies as conducted here and should be based on an evaluation of costs.

## Methods

### Data

The Department of General Internal Medicine, University Hospital of Bern, Switzerland is a tertiary care center providing both in- and outpatient care. Inpatient cases are encoded by medical coding specialists based on the information received from admission and discharge documentation and reports on interventions in the electronic medical record (standardized workflow operatives Medizincontrolling Bern University Hospital). The data pass several quality checks. The coding of inpatient cases is revised annually to ensure high coding standard [32].

The clinical information system provides the inpatients’ medical record. Apart from admission and discharge documentation and reports on interventions it contains information on the whole process of care and treatment.

ICD-10 GM 2014 codes were used to encode main and secondary diagnoses in the original medical statistic data set. Swiss coding guidelines of 2016 were applied as well as the ICD-10-CM Official Guidelines for Coding and Reporting, fiscal year (FY) 2017, Appendix I, Present on Admission Reporting Guidelines for applying the POA indicator [35].

### Study design

Swiss coding guidelines of 2016 [20], the requirements of the medical statistic and the ICD 10 GM [36] were analyzed concerning the potential to encode information on conditions being pre-existent or not (Additional file 2). The results were compared to the resources of encoding the POA indicator referring to the Present on Admission Reporting Guidelines.

The Present on Admission Reporting Guidelines were used to assign the values of the indicator “present at the time of inpatient admission” and “not present at the time of inpatient admission” defined by the rules of the Present on Admission Reporting Guidelines. As the documentation in the medical records was not prepared before admission to meet requirements of POA reporting, the Present on Admission Reporting Guidelines were simplified in respect to the value “unknown” with documentation not being sufficient to report on the time of onset and the value “clinically undetermined” if it is not possible clinically determine whether condition was present on admission or not. The value present-on-admission or not was reported as stated, the value “unknown” and “clinically undetermined” were reported as not present-on-admission.

The selection of the diagnosis groups was based on clinical relevance (diagnoses often associated with complications, with the potential to judge on quality of treatment, not chronic, severe enough to be documented), on the relevance of the use as indicators in performance measurement, on their diversity of the different main diagnosis groups (MDCs), on different clinical situations (i.e. following operation room procedures, lack of nursing, coagulation disorders) and on the results of the survey by the Bern University of Applied Sciences (see Additional files 3 and 4). Defining those groups, a rather broad approach to typical, frequent, clinically relevant, outcome associated and quality related conditions is given, which all might complicate a patient’s treatment at admission or during the stay. Three groups were defined: group 1 “deep vein thrombosis of the lower extremity” = ICD I80.1, I80.2, I80.3 including all sub-codes; group 2 “decubitus ulcer” = ICD L89 including all sub-codes; group 3 “delirium” = ICD F05, F10.4, F11.4, F12.4, F13.4, F14.4, F15.4, F16.4, F17.4, F18.4, F19.4, F43.0, E05.5, including all sub-codes.

Based on the original coding of the medical statistic dataset 2016 of the inpatients’ stays (Department of General Internal Medicine) cases were selected by the previously defined ICD codes. The prevalence of the diagnoses differs. To adjust the number of diagnoses of the previously defined groups, cases were excluded by a random number generator resulting in groups of equal size.

**Fig. 1:**
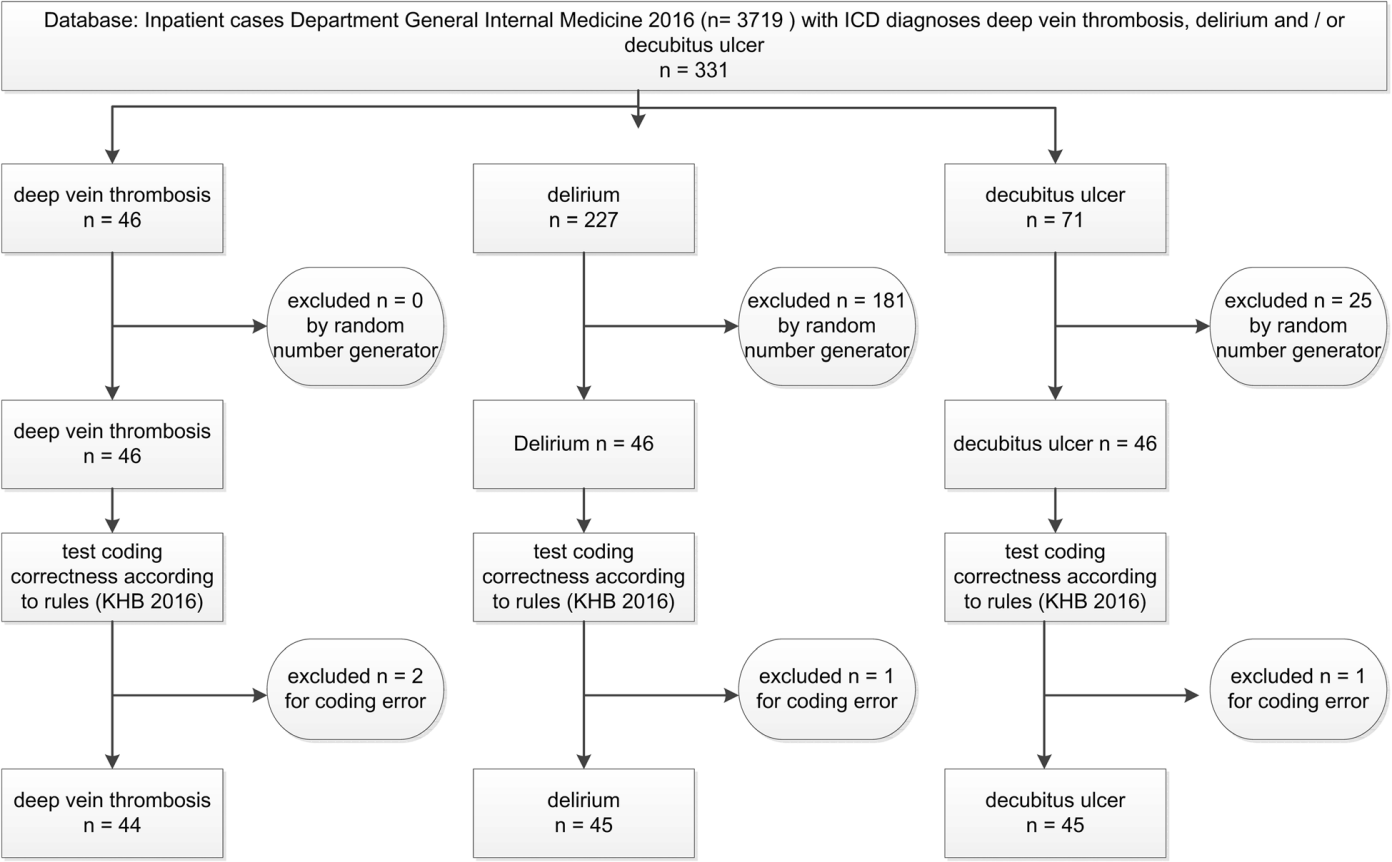
sample flow report

The correctness of the diagnosis code itself was analyzed and cases with coding errors excluded. Figure 1 gives an overview of the sample flow report.

The diagnoses were revised by applying the information present-on-admission by a coding specialist (method 1, standardized workflow and applying POA rules) and by a medical expert (method 2, validating by review of the complete patient’s report). With method 1 a coding expert applied the POA indicator to each of the selected diagnoses according to POA reporting guidelines and the standardized workflow operatives Medizincontrolling Bern University Hospital which is based on the admission and discharge information and intervention reports. With method 2 a medical expert assigned the POA indicator The expert reviewed the complete patient’s report which included the whole process of treatment, thus validating and serving as a Gold Standard. The prevalence of the diagnoses was calculated by selecting all inpatient cases of the hospital’s medical statistic’s dataset using the corresponding ICD codes, (see Additional file 5).

### Analysis

Regarding the information of diagnosis timing the resources of the Swiss medical statistics of hospitals of 2016, of the coding guidelines of 2016 in Switzerland and of the ICD were compared to those of the Present on Admission Reporting Guidelines. The analysis aimed on retrieving information on the diagnosis timing by interpreting the regulations applicable in 2016. When analyzing the medical statistic dataset 2016 either a single encoded diagnosis in one inpatient’s case was defined to carry the information present-on-admission or an explicit complication code as primary diagnosis. Other possibilities to assign the indicator POA with certainty only with the information contained in the Swiss medical statistics of hospitals could be ruled out.

For investigating in the diagnostic quality the values of the indicator present-on-admission of the diagnosis groups and the two methods were compared. Counts of true positive, true negative, false positive, false negative, ratio true positive of total, sensitivity, specificity, positive and negative predictive value, positive and negative likelihood ratio and diagnostic odds ratio were calculated. Cohen’s kappa was calculated to measure the inter-rater reliability of the two methods, kappa values of 0.40–0.59 indicating weak, values of 0.60–0.79 moderate, 0.80–0.90 strong agreement according to literature [37].

Medical Coding Software SAP IS-H Klinischer Arbeitsplatz, Medical Coding Tool ID Diacos, Clinical Data Phoenix CGM, Microsoft Excel 2010, JMP 13.0.0 SAS and umfrageonline.com were used. Prevalence was calculated for all diagnoses groups.

## Results

### Comparing Swiss medical statistics of hospitals 2016 to the present-on-admission reporting guidelines

The coding guidelines of 2016 define rules to code primary and secondary diagnoses and complications. Independently of the time of onset a diagnosis can be coded as a primary diagnosis or secondary. The regulations give the possibility to encode a complication of treatment as primary diagnosis if present at the time of admission and reason for admission. If other diagnoses reveal themselves to be more relevant for the process of treatment, the coding alters. Certain supplementary secondary ICD codes mark a diagnosis in order to carry information on causation or condition. But even these codes are not related to time of onset. With strict interpretation of the existing rules only in two cases a diagnosis can be marked with certainty as present-on-admission: With only one singular diagnosis encoded or in case of a complication of medical treatment, if coded either with a specific code of complication as primary diagnosis or with a supplementary code of causation applied to the primary diagnosis.

No case in dataset 1 met the requirements. All cases were coded with more than one diagnosis and in no case one of the diagnoses of group 1–3 was coded as primary diagnosis connected to a supplementary code of causation. If one of the diagnoses of the defined groups was coded as primary diagnosis, there seemed to be a high probability of the diagnosis being present-on-admission, but with testing the cases on the regulations, no certain designation could be done, see Table 1.

**Table 1:** Present-on-admission information retrievable from the Swiss medical statistics of hospitals 2016^a^ of total of cases at the University Hospital of Bern

	Number of cases	Primary diagnosis of group	Supplementary code indicating complication	Secondary diagnosis coded	POA information retrievable from regulations
Diagnosis group 1: Deep vein thrombosis, lower extremity	44	6	0	yes	no
Diagnosis group 2: Decubitus ulcer and pressure area	45	2	0	yes	no
Diagnosis group 3: Delirium	45	8	0	yes	no
all	134	16	0	yes	no

^a^Variablen der Medizinischen Statistik, Spezifikationen, 1.1.2016, BFS

### Comparison of two coding methods

The total of diagnoses showed different counts in the detection rates at admission. The medical expert (method 2) rated 90 diagnoses (group 1 *n* = 40; group 2 *n* = 28; group 3 *n* = 22) as present-on-admission and the coding specialist (method 1) 95 diagnoses (group 1 *n* = 39; group 2 *n* = 28; group 3 n = 28). The rate of POA positive of the total applied by the coding specialist versus the medical expert was similar, but differed between the diagnosis groups. Different rates could also be shown for the value true positive (Table 2).

**Table 2:** Counts of present-on-admission* and not present-on-admission*, method 1, method 2, for 3 diagnosis groups and all

Diagnosis group 1: Deep vein thrombosis, lower extremity		method 2 medical expert		total
		POA yes	POA no	
method 1 coding expert	POA yes	38	1	39
	POA no	2	3	5
	total	40	4	44
Diagnosis group 2: Decubitus ulcer and pressure area		method 2 medical expert		total
		POA yes	POA no	
method 1 coding expert	POA yes	25	3	28
	POA no	3	14	17
	total	28	17	45
Diagnosis group 3: Delirium		method 2 medical expert		total
		POA yes	POA no	
method 1 coding expert	POA yes	20	8	28
	POA no	2	15	17
	total	22	23	45
all		method 2 medical expert		total
		POA yes	POA no	
method 1 coding expert	POA yes	83	12	95
	POA no	7	32	39
	total	90	44	134

*present-on-admission (= POA “yes”) and not present-on-admission (= POA “no”)

**Fig. 2:**
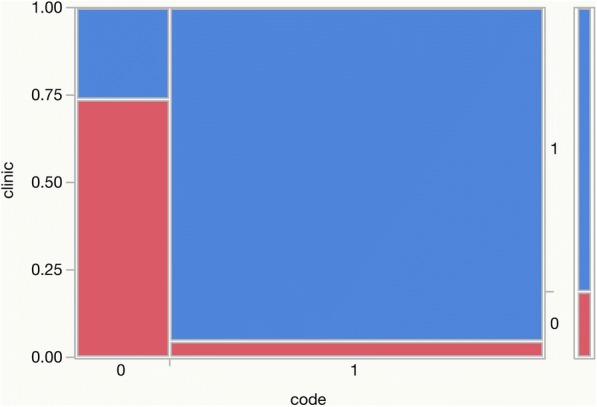
contingency table, present-on-admission (two methods; 0 = POA no; 1 = POA yes; code = POA coded by trained coder; clinic = POA rated by medical expert)

For visual impression a contingency table was set up, see Fig. 2.

The two methods showed a reliability of the information POA of all diagnoses (total of all three groups) of 0.7133 (Cohen’s kappa), but differed with regard to each diagnosis group, as shown in Table 3, including all values concerning the quality of diagnostic test.

**Table 3:** diagnostic value and reliability coding specialist and medical expert^a^

	TP / all	SEN	SPE	PPV	NPV	LR+	LR–	DOR	kappa
Diagnosis group 1: Deep vein thrombosis, lower extremity	0.86	0.95	0.75	0.97	0.60	3.80	0.07	57.00	0.6292
Diagnosis group 2: Decubitus ulcer and pressure area	0.56	0.89	0.82	0.89	0.82	4.96	0.13	38.89	0.7164
Diagnosis group 3: Delirium	0.44	0.91	0.65	0.71	0.88	2.60	0.14	18.75	0.5580
all	0.62	0.92	0.73	0.87	0.82	3.41	0.11	31.62	0.7133

^a^Gold Standard, *Abbreviations TP* true positive, *SEN* sensitivity, *SPE* specificity, *PPV* positive predictive value, *NPV* negative predictive value, *LR+* positive likelihood ratio, *LR*- negative likelihood ratio, *DOR* diagnostic odds ratio

## Discussion

### Comparing Swiss medical statistics of hospitals 2016 to present-on admission reporting guidelines

Analyzing the medical statistic of the selected cases and the resources of the coding guidelines it becomes evident, that without introducing further information the timing of the selected diagnosis cannot be determined. We can state that at present the medical dataset and the ICD do not carry sufficient information on complications and quality of inpatient care. Attributing the indicator POA to ICD diagnoses definitely gives more information on the time of onset of a diagnosis than the present resources of the Swiss medical statistics of hospitals and the Swiss coding guidelines of 2016. As the ICD catalogue is very limited itself concerning diagnosis timing, even further elaborated coding guidelines would not meet the need of an indicating information on each diagnosis.

### Comparison of two coding methods discussion

Coding the POA indicator identified diagnoses which were pre-existent with a sufficient to good diagnostic quality but only moderate reliability. The inter-rater reliability was weak in group 3 “delirium”, but moderate in group 1 “deep vein thrombosis lower extremity” and group 2 “decubitus ulcer”. As the rating methods differed with regard to the documents used (method 1 based on the admission and discharge information and intervention reports; method 2 review of the complete patient’s report which included the whole process of treatment), the reason for this difference could be a loss of information. However, the difference of the ratio of positively rated diagnoses of all (POA/all) does not support this explanation. The medical reports had not been previously prepared for POA reporting. The study was conducted retrospectively and there was no training of documenting diagnoses at admission targeting on POA reporting. To avoid an over-reporting concerning present-on-admission and to facilitate the application of the indicator, the value “unknown” and “clinically undetermined” were reported as not present-on-admission (simplified POA Reporting Guidelines). Further investigations following this study with specifically prepared medical reports and instructions might achieve a complete database including the values “unknown” and “clinically determined” with a higher reliability. Nevertheless, the differences of the diagnosis groups might partly be due to the clinical relevance and explicitness of symptoms. The diagnosis delirium, decubital ulcer and thrombosis show specific manifestation time frames, specific severity of symptoms and a specific detectability. Also, the different diagnosis might be specifically susceptible to become a complication of inpatient treatment, with different observed incident rates.

The data supports the clinical situation with different counts of TP and TN, and different ratio of positively rated diagnoses of all (POA/all; TP/all) between the diagnosis groups with highest proportions detected in group 1 “deep vein thrombosis lower extremity” and lowest in group 3 “delirium”.

The diagnostic value calculated for all diagnoses is sufficient and meets the specification of diagnostic tests. However, the values of sensitivity and specificity as well as positive and negative predictive value differed between the diagnosis groups. We presume that the successful interpretation of symptoms as documented in the medical record following the POA Reporting Guideline “If the final diagnosis contains a possible, probable, suspected, or rule out diagnosis, and this diagnosis was based on signs, symptoms or clinical findings that were not present on admission, assign no “N” (i.e. not present-on-admission)” and “If the final diagnosis contains a possible, probable, suspected, or rule out diagnosis, and this diagnosis was based on signs, symptoms or clinical findings suspected at the time of inpatient admission, assign yes “Y.” (i.e. present-on-admission)” depends on the clinical impact of the diagnosis itself. As mentioned before, it influences the documentation in the medical report.

With regard to the diagnostic quality and the reliability we conclude that within the process of ICD coding designating diagnoses as being present-on-admission contains sufficient information to be used as an indicator (e.g. risk-adjustment). However, the use of POA as an indicator should be limited at present to specified groups of cases and should be evaluated for every target diagnosis in order to ensure validity. Considering pay-for-performance systems which aim on calculating a reimbursement rate for individual inpatient cases by use of the POA information, the only moderate to weak reliability must be improved [23, 26]. The official Present on Admission Reporting Guidelines encourage coders to “query the providers when the documentation is unclear” [35]. This explicit demand reveals the problems of designating the information. With this study, we omitted queries (there was no query of the documenting provider) and assigned the value “unknown” and “clinically undetermined” to not present-on-admission, in order to give an unambiguous impression of the current resources of POA reporting with no preparation of documentation beforehand and second to elaborate the inter-rater reliability. The inter-rater reliability can be improved by queries, which should be practiced when questions of accuracy of coding occur. But at this point using the two role-bound methods we tried to give an impression on the different results without interfering communication. The values suggest that monitoring quality of treatment and complexity of patient groups retrospectively by use of POA as an indicator is possible. However, with a weak reliability it should not be used for prospective quality measures or reimbursement of individual inpatient cases so far.

As the correct application of the indicator POA depends on a thorough examination at admission, on the quality and completeness of documentation in the medical report, on clear definitions and coding regulations and on the medical expertise of the coding staff, efforts in these fields should be undertaken to prepare for POA reporting in future [22, 27, 29–31]). Although coding the patient’s record in hindsight influences the judgement upon symptoms at admission, the aim of a valid retrospective monitoring of the timing of diagnoses can be achieved. At discharge with the patients’ record completed, full information concerning all symptoms and diagnoses is accessible to the coder and thus leads to a verifiable decision. Using this method of applying the POA indicator enables institutions to evaluate retrospectively.

Also, as the prevalence of the underlying conditions and diagnoses varies and validity and reliability on the individual patient’s level could be questioned with insufficient reliability, the indicator should be embedded in more sophisticated approaches as for example the Australian automated Classification of Hospital Acquired Diagnoses (CHAD) [12, 13] or the Potentially Preventable Complications (PPC) [38] classification system which includes risk adjustment. The different prevalence has a relevant impact on the consequences resulting from false positive and false negative cases and from different values of the diagnoses groups, e.g. the diagnosis group “delirium” shows the highest prevalence but lowest PPV, (see Additional file 5).

### Strengths and limitations

The strength lies in the quality of data, which is highly standardized by requirements of the Swiss medical statistics of hospitals and undergoes quality checks after coding. Therefore, the data is comparable to data of other tertiary care centers in Switzerland. This study is the first using data from Switzerland to analyze the diagnostic value of the present-on-admission indicator and one of the few worldwide calculating reliability in medical coding. It gives an incentive to further investigations, which are needed before implementing POA reporting in different European countries. Limitations of the study are the retrospective design with the documentation of the medical report not prepared for POA reporting as well as the small number of diagnoses especially as the diagnosis groups showed different results. As documentation in medical records is not standardized between the providers, the results might not be comparable [39].

## Conclusions

By these findings, in accordance with the development of requirements of administrative data in Switzerland and other countries, it can be concluded, that an implementation of the variable present-on-admission into the medical dataset should be evaluated.

The implementation would enable risk-adjustment, refined disease surveillance, research, case-mix costing and reports on quality indicators, for in-house, national and international analysis. However, these results as well as studies especially from the US point out, how important an assessment of the diagnostic value of the indicator POA and each diagnosis group is. Only when considering the validity for each diagnosis group the results of a future POA reporting should be used for monitoring and measures. The insufficient reliability reveals a further need to improve documentation and coding standards for the POA indicator in order to achieve high values on the individual patient’s level. The results show a need of standardization of documentation. To retrieve sufficient information from the documentation meeting the standard of a medical expert and to rise the reliability of coding, the diagnosis timing should be documented explicitly.

However, with regard to likelihood ratio the quality of the test seems sufficient as an indicator on population level. The differences of the results between the diagnoses groups emphasize the need to evaluate more diagnoses groups for diagnostic quality, covering more MDCs and different clinical conditions (i.e. following operation room procedures, lack of nursing, coagulation disorders).

The study shows that with rising costs in health care and rising requirements of quality of inpatient care the indicator “present-on-admission” offers the opportunity to retrieve essential information on diagnosis timing from routinely collected health data. A sensible approach would be a selection of diagnoses which are documented in literature serving as verified quality indicators (e.g. pressure ulcus) [29, 30]. In many European health care systems encoding all inpatient cases is mandatory for national statistics and/or reimbursement (e.g. Germany, Switzerland). Coding the indicator POA for a certain set of diagnoses would mean a moderate additional effort. However, deriving information about the onset of diagnoses from the medical records demands detailed notes and a highly standardized documentation for all records. This demand meets legal requirements concerning liability, but will cause substantial effort.

If introduced with care, POA can connect analysis and research, resource allocation and quality of care based on standardized and comparable national and international administrative data.

## Additional files

Additional file 1: Survey Bern University of Applied Sciences. (DOCX 16 kb)

Additional file 2: Swiss coding guidelines 2016 and requirements of the Swiss medical statistics of hospitals*: regulations concerning complications and diagnosis timing. (DOCX 15 kb)

Additional file 3: Survey Bern University of Applied Sciences diagnosis groups of interest, diagnosis to be reported. (DOCX 15 kb)

Additional file 4: ICD codes for selection of diagnosis groups 1, 2 and 3. (DOCX 15 kb)

Additional file 5: Prevalence of diagnoses groups at the Insel Gruppe 2017. (DOCX 15 kb)

## Abbreviations

ANQ: Nationaler Verein für Qualitätsentwicklung in Spitälern
BAG: Federal Office of Health Switzerland (Bundesamt für Gesundheit)
BFS: Federal Office of Statistics Switzerland (Bundesamt für Statistik)
CMS: Centers for Medicare and Medicaid Services
DOR: Diagnostic odds ratio
FMH: Swiss Medical Association (Foederatio Medicorum Helveticorum)
FY: Fiscal year
GEF: Gesundheits- und Fürsorgedirektion Kanton Bern
H plus: Association H+ Die Spitäler der Schweiz
ICD: International Statistical Classification of Diseases
ICD-10 GM 2014: ICD-10 German Modification 2014
IQM: Initiative Qualitätsmedizin
KEK: Ethics Committee of the Canton Bern
KHB: Swiss Coding Guideline
LR +: Positive likelihood ratio
LR-: Negative likelihood ratio
MDC: Main diagnosis groups
MS: Medical statistic (Swiss medical statistics of hospitals)
N: Indicator POA no
NPV: Negative predictive value
OECD: Organisation for Economic Co-operation and Development
POA: Present-on-admission
PPC: Potentially Preventable Complications
PPV: Positive predictive value
SEN: Sensitivity
SPE: Specificity
Swiss DRG: Swiss Diagnosis Related Groups
TP: Total positive
Unifin: Gruppe der Finanz- und Tarifverantwortlichen der Universitätsspitäler (group of head of financial departments university hospitals switzerland)
US: United States of America
WHO: Word Health Organization
Y: Indicator POA yes
ZIM: Zentrum für Informatik und wirtschaftliche Medizin
