# Supplementary material for: Diagnostic value and reliability of the present-on-admission indicator in different diagnosis groups: pilot study at a Swiss tertiary care center
Source: BMC Health Serv Res. 2019 Jan 9;19:23. doi: 10.1186/s12913-018-3858-3 (PMC6327414; doi:10.1186/s12913-018-3858-3)
Supplement: Supplementary file 1 — Survey Bern University of Applied Sciences. (DOCX 16 kb) [file 12913_2018_3858_MOESM1_ESM.docx]

| **Additional file 1**: survey Bern University of Applied Sciences POA 2016 | | | |
| --- | --- | --- | --- |
|  | yes | no | undecided |
| Do you believe that the quality indicators for treatment of inpatient cases as currently collected in Switzerland, are sufficient to assess the quality of treatment of different hospitals at national level? | 3 | 12 | 1 |
| Do you believe that the quality indicators for treatment of inpatient cases as currently collected in Switzerland, are sufficient to assess the quality of treatment of different hospitals at international level? | 2 | 12 | 2 |
| Do you consider the possibilities to represent complications and comorbidities by means of ICD coding and the medical data set sufficient to assess the quality of treatment of different hospitals at national level? | 4 | 9 | 3 |
| Do you consider the possibilities to represent complications and comorbidities by means of ICD coding and the medical data set sufficient to assess the quality of treatment of different hospitals at international level? | 6 | 7 | 3 |
| Do you consider the resources of ICD coding of complications and comorbidities and the medical data set sufficient to prevent unintended incentives in reimbursement? | 1 | 9 | 6 |
| Do you consider the currently used quality indicators sufficient to prevent unintended incentives in reimbursement? | 0 | 15 | 1 |
| Are you familiar with the indicator "present on admission" (POA) as a supplementary information on diagnoses of inpatient cases? | 11 | 6 | 0 |
| Is your clinic / institution interested in inhouse data on diagnosis timing? | 10 | 1 | 4 |
| Is your clinic / institution interested in national data on diagnosis timing? | 12 | 1 | 2 |
| Is your clinic / institution interested in national data on diagnosis timing by using the indicator "present on admission" POA? | 12 | 1 | 2 |
| Do you consider the indicator "present on admission" POA sufficient for inhouse monitoring of the quality of inpatient treatment? | 13 | 2 | 0 |
| Do you consider the indicator "present on admission" POA sufficient for the monitoring of the quality of inpatient treatment at national level? | 14 | 1 | 0 |
| Do you consider the indicator "present on admission" POA sufficient for the monitoring of the quality of inpatient treatment at international level? | 14 | 1 | 0 |
| Do you consider the indicator "present on admission" POA sufficient to prevent unintended incentives in reimbursement? | 5 | 6 | 4 |
| Do you consider the indicator "present on admission" POA sufficient to improve the current reimbursement system SwissDRG? | 11 | 0 | 0 |
| If so, would you prefer to collect data on all ICD diagnoses? | 2 | 11 | 2 |
| Would it be sensible to collect the data "present on admission" POA on all ICD diagnoses with exception of a defined number (e.g.congenital defects)? | 6 | 5 | 4 |
| Would it be sensible to collect the data "present on admission" POA only on previously defined ICD diagnoses (blacklist)? | 12 | 0 | 3 |
| *19 participants: health providers (H+, Unifin), BFS, BAG, Gesundheits- und Fürsorgedirektion Kanton Bern (GEF), provider of the present patient classification system (SwissDRG AG), software companies, Zentrum für Informatik und wirtschaftliche Medizin (ZIM), health insurers (santesuisse and curafutura), Nationaler Verein für Qualitätsentwicklung in Spitälern und Kliniken (ANQ) , Swiss Medical Association (FMH), in-house clinicians and key persons of quality and data management of the Bern University Hospital | | | |
