# Supplementary material for: Diagnostic value and reliability of the present-on-admission indicator in different diagnosis groups: pilot study at a Swiss tertiary care center
Source: BMC Health Serv Res. 2019 Jan 9;19:23. doi: 10.1186/s12913-018-3858-3 (PMC6327414; doi:10.1186/s12913-018-3858-3)
Supplement: Supplementary file 2 — Swiss coding guidelines 2016 and requirements of the Swiss medical statistics of hospitals*: regulations concerning complications and diagnosis timing. (DOCX 15 kb) [file 12913_2018_3858_MOESM2_ESM.docx]

| **Additional file 2:** Swiss coding guidelines 2016 (KHB) and requirements of the medical statistic*: regulations concerning complications and diagnosis timing | | |
| --- | --- | --- |
|  |  |  |
| regulation number | object | rule |
| KHB G52c | primary diagnosis | the condition, diagnosed at the end of the  episode of health care, primarily responsible for the patient’s need for treatment or investigation, no reason for admission rule |
| KHB G53b | supplementary code | causation and condition, no diagnosis timing |
| KHB G54c | secondary diagnosis | no diagnosis timing |
| KHB D06c | sequelae | no diagnosis timing  ICD codes imply with high probability information present-on-admission |
| KHB D12c | complications | If only reason for admission primary diagnosis; if complication of actual medical treatment secondary diagnosis; can be interpreted with high probability as being present-on-admission or not if specific complication codes and supplementary codes are coded |
| KHB D16c | complication at readmission 18 days | secondary diagnosis |
| MS 1.6.V02, 4.2.V020 | supplementary code | no diagnosis timing |
| MS 1.6.V03 –V10, 4.2.V03- 51 | supplementary code | no diagnosis timing |
| *Medizinisches Kodierungshandbuch, Der offizielle Leitfaden der Kodierrichtlinien in der Schweiz, Version 2016, BFS; Variablen der Medizinischen Statistik (MS) , Spezifikationen, 1.1.2016, BFS | | |
