# Supplementary material for: Diagnostic value and reliability of the present-on-admission indicator in different diagnosis groups: pilot study at a Swiss tertiary care center
Source: BMC Health Serv Res. 2019 Jan 9;19:23. doi: 10.1186/s12913-018-3858-3 (PMC6327414; doi:10.1186/s12913-018-3858-3)
Supplement: Supplementary file 3 — Survey Bern University of Applied Sciences diagnosis groups of interest, diagnosis to be reported. (DOCX 15 kb) [file 12913_2018_3858_MOESM3_ESM.docx]

| **Additional file 3:** survey Bern University of Applied Sciences diagnosis groups of interest, diagnosis to be reported | | | | | |
| --- | --- | --- | --- | --- | --- |
|  | | | | | |
| Which category of diagnoses would be most interesting for you when thinking of the indicator „present-on-admission“ POA? | | | | | |
| Category of diagnoses | thrombosis | embolism | infections | decubital ulcer | hemorrhagic diathesis |
| Counts participants | 9 | 7 | 9 | 9 | 3 |
|  |  |  |  |  |  |
| Category of diagnoses | stroke | sepsis | pulmonary edema | atelectasis | anemia |
| Counts participants | 7 | 9 | 4 | 3 | 4 |
| *19 participants: health providers (H+, Unifin), BFS, BAG, Gesundheits- und Fürsorgedirektion Kanton Bern (GEF), provider of the present patient classification system (SwissDRG AG), software companies, Zentrum für Informatik und wirtschaftliche Medizin (ZIM), health insurers (santesuisse and curafutura), Nationaler Verein für Qualitätsentwicklung in Spitälern und Kliniken (ANQ) , Swiss Medical Association (FMH), in-house clinicians and key persons of quality and data management of the Bern University Hospital; 2016 | | | | | |
