# Supplementary material for: Diagnostic value and reliability of the present-on-admission indicator in different diagnosis groups: pilot study at a Swiss tertiary care center
Source: BMC Health Serv Res. 2019 Jan 9;19:23. doi: 10.1186/s12913-018-3858-3 (PMC6327414; doi:10.1186/s12913-018-3858-3)
Supplement: Supplementary file 4 — ICD codes for selection of diagnosis groups 1, 2 and 3. (DOCX 15 kb) [file 12913_2018_3858_MOESM4_ESM.docx]

| **Additional file 4:** ICD codes for selection of diagnosis groups 1, 2 and 3 | | |
| --- | --- | --- |
|  |  |  |
|  | **ICD 10 GM 2014 codes** | **ICD 10 GM 2014 text** |
|  |  |  |
| **Diagnosis group 1: Deep vein thrombosis, lower extremity** | I80.1 | Thrombosis, phlebitis and thrombophlebitis of femoral vein |
|  | I80.2* | Thrombosis, phlebitis and thrombophlebitis of other deep vessels of lower extremities |
|  | I80.3 | Thrombosis, phlebitis and thrombophlebitis of lower extremities, unspecified |
|  |  |  |
| **Diagnosis group 2: Decubitus ulcer and pressure area** | L89.0* | Stage I decubitus ulcer and pressure area |
|  | L89.1* | Stage II decubitus ulcer |
|  | L89.2* | Stage III decubitus ulcer |
|  | L89.3* | Stage IV decubitus ulcer |
|  | L89.9* | Decubitus ulcer and pressure area, unspecified |
|  |  |  |
| **Diagnosis group 3: Delirium** | F05* | Delirium, not induced by alcohol and other psychoactive substances |
|  | F10.4 | Mental and behavioural disorders due to use of alcohol, Withdrawal state with delirium |
|  | F11.4 | Mental and behavioural disorders due to use of opioids, Withdrawal state with delirium |
|  | F12.4 | Mental and behavioural disorders due to use of cannabinoids, Withdrawal state with delirium |
|  | F13.4 | Mental and behavioural disorders due to use of sedatives or hypnotics, Withdrawal state with delirium |
|  | F14.4 | Mental and behavioural disorders due to use of cocaine, Withdrawal state with delirium |
|  | F15.4 | Mental and behavioural disorders due to use of other stimulants, including caffeine, Withdrawal state with delirium |
|  | F16.4 | Mental and behavioural disorders due to use of hallucinogens, Withdrawal state with delirium |
|  | F17.4 | Mental and behavioural disorders due to use of tobacco, Withdrawal state with delirium |
|  | F18.4 | Mental and behavioural disorders due to use of volatile solvents, Withdrawal state with delirium |
|  | F19.4 | Mental and behavioural disorders due to multiple drug use and use of other , Withdrawal state with delirium psychoactive substances |
|  | F43.0 | Acute stress reaction |
|  | E05.5 | Thyroid crisis or storm |
| “*” = all sites, all subcategories | | |
