# Supplementary material for: Diagnostic value and reliability of the present-on-admission indicator in different diagnosis groups: pilot study at a Swiss tertiary care center
Source: BMC Health Serv Res. 2019 Jan 9;19:23. doi: 10.1186/s12913-018-3858-3 (PMC6327414; doi:10.1186/s12913-018-3858-3)
Supplement: Supplementary file 5 — Prevalence of diagnoses groups at the Insel Gruppe 2017. (DOCX 15 kb) [file 12913_2018_3858_MOESM5_ESM.docx]

| **Additional file 5:** prevalence in % of diagnoses groups inpatient cases at the Insel Gruppe 2016 | | | |
| --- | --- | --- | --- |
|  | 1: Deep vein thrombosis, lower extremity | 2: Decubitus ulcer and pressure area | 3: Delirium |
| Insel Gruppe | 0.48 | 0.79 | 2.51 |
| Department of General Internal Medicine, Inselspital | 1.10 | 1.70 | 5.45 |
